# Depression and anxiety in early adulthood: consequences for finding a partner, and relationship support and conflict

**DOI:** 10.1017/S2045796020000530

**Published:** 2020-07-15

**Authors:** L.S. Leach, P. Butterworth

**Affiliations:** 1 National Centre for Epidemiology and Population Health (NCEPH), Research School of Population Health, The Australian National University, Canberra, ACT, Australia; 2 Centre for Research on Ageing, Health & Wellbeing, Research School of Population Health, The Australia National University, Canberra, ACT 0200, Australia; 3 Melbourne Institute of Applied Economic and Social Research, The University of Melbourne, Melbourne, VIC, Australia

**Keywords:** Anxiety, depression, prospective, relationship quality, young adults

## Abstract

**Aims:**

Mental health problems in early adulthood may disrupt partner relationship formation and quality. This prospective study used four waves of Australian data to investigate the effects of depression and anxiety in early adulthood on the quality of future partner (i.e. marriage or cohabiting) relationships.

**Methods:**

A representative community sample of Australian adults aged 20–24 years was assessed in 1999, 2003, 2007 and 2011. Analyses were restricted to those who at baseline had never entered a marriage or cohabiting relationship with no children (*n* = 1592). Associations were examined between baseline depression and anxiety levels (using the Goldberg Depression and Anxiety scales) and (a) future relationship status and (b) the quality of marriage or cohabiting relationships recorded at follow-up (up to 12 years later) (partner social support and conflict scales).

**Results:**

Depression in early adulthood was associated with never entering a partner relationship over the study period. For those who did enter a relationship, both depression and anxiety were significantly associated with subsequently lower relationship support and higher conflict. Supplementary analyses restricting the analyses to the first relationship entered at follow-up, and considering comorbid anxiety and depression, strongly supported these findings.

**Conclusions:**

Depression and anxiety in early adulthood is associated with poorer partner relationship quality in the future. This study adds to evidence showing that mental health problems have substantial personal and inter-personal costs. The findings support the need to invest in prevention and early intervention.

## Introduction

Substantial epidemiological research has sought to identify and quantify the extensive functional, social and economic consequences of psychiatric disorders. Global Burden of Disease research showed that in 2010 mental and substance use disorders accounted for 7.4% (6.2–8.6%) of all disability-adjusted life-years worldwide (Whiteford *et al*., 2013). Although studies quantifying the overall burden of mental disorders using macro-level indicators are critical, nuanced research is needed in tandem to demonstrate the social and relational consequences of mental health problems – particularly the most common mental health problems, depression and anxiety.

Studies have demonstrated the wide range of consequences when depression and anxiety begin early in life. This research shows that depression and anxiety experienced during adolescence or early adulthood can disrupt the achievement of normative milestones – including high school completion (Butterworth and Leach, 2017 – psychological distress; Kessler *et al*., 1995 – anxiety and depressive mood disorder) and gaining employment (Fergusson and Woodward, 2002 – major depression). Studies have also investigated the impact of early onset mental health disorders on the timing and stability of partner relationships (i.e. married or cohabiting) and childbearing (Forthofer *et al*., 1996; Kessler *et al*., 1997, 1998). Retrospective (cross-sectional) data from the National Comorbidity Survey in the USA has shown that anxiety, depressive, conduct and substance use disorders are associated with early first marriage (Forthofer *et al*., 1996), teenage parenthood (Kessler *et al*., 1997) and subsequent separation and divorce (Kessler *et al*., 1998). However, little prospective epidemiological research has specifically assessed the impacts of *early adulthood* depression and anxiety on the *quality* of future partner relationships. This is the primary focus of the current study.

Lower depression symptomology is associated with better relationship quality (Weissman, 1987; Dehle and Weiss, 1998; Whisman, 1999; Kiecolt-Glaser and Wilson, 2017). Longitudinal research investigating the temporal association between depression and relationship quality has adopted both a causation perspective (i.e. relationship quality predicts poor mental health over time) and a selection perspective (i.e. poor mental health select individuals into poor quality relationships) (Kim and McKenry, 2002), and indicates that the links are bi-directional (Kiecolt-Glaser and Wilson, 2017). For example, Davila *et al*. (2003) showed a bidirectional relationship between marital satisfaction and depression symptoms over a 4-year period. Najman *et al*. (2014) followed 2971 women first interviewed during pregnancy over 21 years and observed a bidirectional association between poor marital quality and depression. Although depression has been the focus of this research, studies on anxiety similarly indicate that anxiety symptomology and/or disorder is associated with poorer relationship quality (see McLeod, 1994; Whisman, 1999 and Leach *et al*., 2013 for examples of large population based studies; see Dehle and Weiss, 2002 and Zaider *et al*., 2010 for smaller couple-focused and qualitative studies).

Although there is substantial research investigating depression and anxiety and poor relationship quality over time, the extent to which experiences *specifically in early adulthood* (prior to engagement in married or cohabiting relationships) go on to influence future partner relationship quality remains relatively unexplored. Early adulthood is a critical and complex time of life. The transition to adulthood appears to be extending beyond adolescence – as young people struggle to negotiate education, carer and family formation goals (Settersten *et al*., 2008). Depression and anxiety at this time likely has adverse consequences for future relationships. In one of the only prospective studies available (*n* = 1700), diagnostic depression in adolescence predicted lower relationship quality in early adulthood for both women and men (Gotlib *et al*., 1998). However, this study is limited by a short follow-up period (only until mid-20s), and the impacts of anxiety were not considered.

The current used four time-points of data (spanning 12 years) from an Australian population-based cohort study to investigate the consequences of depression and generalised anxiety during early adulthood (prior to first marriage or cohabiting relationship) for both: (a) future relationship status and (b) future relationship quality. We control for a range of potential confounders associated with depression and/or anxiety including gender (McLean *et al*., 2011; Salk *et al*., 2017), age (Jorm, 2000), education (Butterworth and Leach, 2017), employment (Paul and Moser, 2009), financial hardship (Kiely *et al*., 2015), alcohol use (Rodgers *et al*., 2000), smoking (Lasser *et al*., 2000) and physical functioning (Ohrnberger *et al*., 2017). The main analyses explore the impact of baseline anxiety and depression on future relationship quality using all time-points where data are available. Supplementary analyses are also conducted to confirm the impacts on relationship quality in the ‘first’ relationship entered to omit the effects of multiple relationships.

## Methods

### Participants and procedure

Data were from four time-points of the Personality and Total Health (PATH) Through Life project, a large community-based cohort study based at The Australian National University that measures physical health, mental health, cognitive and personality characteristics across the lifespan. Initially, potential participants were selected at random from the electoral rolls of Canberra ACT and Queanbeyan NSW Australia, within three age cohorts: 20–24 years, 40–44 years and 60–64 years. Follow-up data have been collected at approximately four yearly intervals and to date, four waves of data have been collected (see Anstey *et al*., 2011).

The current paper focuses on the youngest 20s cohort. In this cohort, 2404 respondents (48.3% male) completed the baseline assessment (February 1999–April 2000) representing 58.6% of the invited population. For the baseline and follow-up data collection at wave 2 (*m* = 4.10 years later in April 2003–June 2004) and wave 3 (*m* = 3.97 years later in April 2007–April 2008), respondents were interviewed by a trained interviewer. Participants completed self-report measures and the interviewer additionally administered face-to-face physical and cognitive tests. For wave 4 follow-up (*m* = 4.06 years later in May 2011–May 2012), all participants were invited to complete the self-report measures online, and a randomly selected subsample of 580 participated in a face-to-face interview with additional physical and cognitive tests. All data used in the current study were from the self-reported measures at each wave.

The sample was initially restricted to 1592 participants (54.6% male) who at baseline (aged 20–24) had never married, did not live with a partner and did not have children. The first set of analyses examined the effect of baseline generalised anxiety and depression levels on future relationship status – comparing those who *never* reported entering a relationship (*n* = 449) with those who entered a relationship at follow-up (*n* = 1143). The 1030 participants who reported being in a relationship at one of the follow-up periods were included in a second set of analyses focused on relationship quality (wave 2: *n* = 612, wave 3: *n* = 809, wave 4: *n* = 576; 1997 observations). Table 1 shows the number of participants who reported their first relationship at each wave, continued to report not being in a relationship, reported multiple relationships and dropped-out of the study. There was little item-missing data (<1% on any variable). Therefore, no data imputation was conducted.

**Table 1.** Partner relationship status over the three follow-up waves (time-points of data collection)

	W2 (24–28)	W3 (28–32)	W4 (32–36)	Total
Never in a relationship	630 (38%) 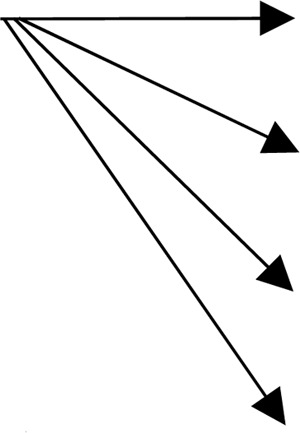	284 (45%) 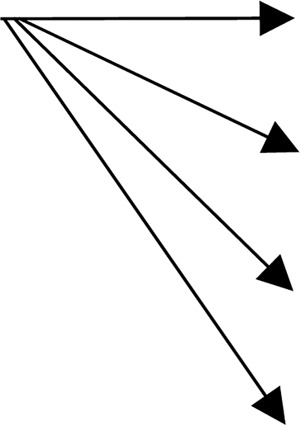	108 (38%)^a^	449^a^
In first relationship	522 (33%)^b^	212 (36%)^b^	49 (17%)^b^	783^b^
Have been in multiple relationships	266 (17%)^c^	67 (11%)^c^	27 (10%)^c^	360^c^
Dropped out of study	174 (11%)^a^	67 (11%)^a^	100 (35%)^a^	–
Total in sample	1592			1592

*Note*: At W1, everyone in the sample was not in a partner relationship and had never been married.

a 449 recorded as ‘never in a relationship’ at the last time they were interviewed (i.e. they may have subsequently dropped out of the study).

b 783 recorded as in their first relationship at one of the three follow-up time-points.

c 360 recorded as having been in multiple relationships at the time of interview.

### Measures

*Depression and anxiety* were measured at all waves using the Goldberg Depression and Anxiety Scales (Goldberg *et al*., 1988). Each scale contains nine symptom-related items (yes = 1; no = 0) summed to yield scale scores ranging from 0 to 9. The scales have been found to effectively detect elevated levels of depression and generalised anxiety in community samples (Mackinnon *et al*., 1994; Kiely and Butterworth, 2015). Although the correlation between the two Goldberg Scales is high (*r* = 0.71, *p* < 0.001 in this sample), a two factor model with separate depression and anxiety dimensions is supported (Christensen *et al*., 1999). Both scales have good internal reliability in the PATH sample (depression: *α* = 0.81; anxiety *α* = 0.78). Both scales were rescaled to aid in interpretation of the regression coefficients – such that a one-point increase on the scales represents the difference between the 25th and the 75th percentiles on the distribution (i.e. a score of ‘0’ represents (low) scores on the 25th percentile and a score of ‘1’ represents (high) scores on the 75th percentile). Binary scores representing likely depression and generalised anxiety disorder diagnosis were also calculated based on validated cut-points assessed against the CIDI (i.e. a score of ⩾5 on the depression scale and ⩾7 on the anxiety scale (Kiely and Butterworth, 2015)) to describe the proportion of the sample with likely depression and/or generalised anxiety disorder, and in supplementary analyses exploring the impact of comorbidity.

*Relationship status* was assessed at each wave using an item that asked: are you currently in a relationship with someone? Responses were: 1 – ‘Yes, living with the person you are married to’, 2 – ‘Yes, living with a partner (but not married to them)’, 3 – ‘Yes, in a relationship with someone but not living with them’ and 4 – ‘No, not in a relationship with anyone’. This item was recoded to represent a binary indicator of relationship status where 1 represented ‘in a marriage/cohabiting relationship’ (i.e. responses 1 or 2) and 0 represented ‘not in a marriage/cohabiting relationship’ (i.e. responses 3 or 4). An item also asked at each wave ‘How many times have you been married or lived with a partner?’ A measure of partner relationship status over the follow-up period was created where 1 represented ‘observed in first marriage/cohabiting relationship’, 0 represented ‘never observed in a marriage/cohabiting relationship’, and 2 represented ‘observed in multiple marriages/cohabiting relationships’.

*Social support and conflict* within the partner relationship were assessed using items developed and validated by Schuster *et al*. (1990). Perceived positive support was assessed using five items, including ‘How often does your partner understand the way you feel about things?’ Negative interactions or conflict were assessed using five items, including ‘How often do you have an unpleasant disagreement with your partner?’ Possible responses for both sets of items were: 0 – ‘Never’, 1 – ‘Rarely’, 2 – ‘Sometimes’ and 3 – ‘Often’. The conflict items did not ask about physical conflict, but instead focused on disagreement and tension. Total scale scores ranged from 0 to 15. Scale scores were standardised to provide *z*-scores to assist with interpretation. Preliminary factor analyses confirmed the existence of two separate factors representing support and conflict. Measures of relationship support and conflict experienced in the *first* marriage or *de facto* relationship observed were also constructed (i.e. data were recorded only once for participants in the first relationship entered and was then censored).

#### Covariates

Socio-economic covariates included gender, age, years of education and employment status. A measure of financial hardship was generated based on one item: ‘Have you or your family had to go without things you really needed in the last year because you were short of money?’ Respondents were classified as having financial hardship (1) if they responded ‘yes, often’ or ‘yes, sometimes’ to this item (*v*. ‘no’). A binary measure of current smoking was included, as was the alcohol use disorders identifications test (AUDIT) (Saunders *et al*., 1993). Participants were classified into one of three alcohol use categories based on the National Health and Medical Research Council (2001) guidelines: (a) non-drinkers or occasional drinkers (⩽monthly), (b) moderate drinkers (<28 standard drinks per week for men and <14 for women) or (c) hazardous or harmful drinkers (⩾28 drinks per week for men and ⩾14 for women) (NHMRC, 2001). Given the comorbidity between mental and physical health problems, the short-from physical health summary (SF-12) was also included as measure of physical functioning (Ware *et al*., 1996). A variable representing whether each participant dropped out of the study was also constructed (0, 1).

### Statistical analyses

Descriptive statistics for the baseline analysis sample were initially calculated. A series of logistic regression models were used to examine the association between baseline levels of depression and anxiety (at age 20–24) and odds of subsequently entering (or not entering) a partner relationship during the study period (12 years). These models adjusted for attrition (i.e. ‘dropped-out’ *v*. ‘never dropped-out’ of the study), given attrition was significantly associated with never being in a relationship (*χ*^2^ = 177.53, *p* < 0.001).

Analyses used the full panel of data (four waves) in random effects models to examine the effect of baseline mental health on future relationship support and conflict at the follow-ups. The analyses adjusted for all covariates across the three-time points (i.e. time varying), including the wave at which data were recorded. In a final model, the analyses included a variable representing current (or follow-up) depression and anxiety symptom levels to further ascertain the independent contribution of *baseline* mental health. Potential gender differences were evaluated by the inclusion of an interaction term, however as this term was not significantly associated with either relationship status at follow-up, or levels of relationship support or conflict, all models included both men and women. Although the social support and conflict measures (outcomes) were skewed (with respondents more likely to report high levels of social support and low levels of conflict), no transformations were undertaken in the main analyses as the large sample size was sufficiently robust to violations of normality (Lumley *et al*., 2002). For all tests significance was set at *p* < 0.05. Data were analysed using STATA SE version 15.

A series of supplementary analyses were used to aid in the interpretation of the findings and to test their robustness. First, analyses examined the impact of baseline depression and anxiety on levels of partner support and conflict experienced in the *first* marriage or cohabiting relationship recorded at follow-up to omit the potential influence of multiple relationships (i.e. outcome data were recorded in the first relationship entered and was then censored). These models adjusted for the time-point at which the first relationship was entered. Second, the relationship quality outcomes were transformed to explore improving the skewed distribution. Final analyses assessed the impact of comorbidity (experiencing depression or anxiety concurrently) in association with subsequent relationship quality.

## Results

### Descriptive analyses – characteristics of the sample

The average depression score at baseline for the full sample was 2.6 for males and 3.0 for females, and the average anxiety score was 3.2 for males and 4.3 for females. Using the validated cut-points for the Goldberg scales at baseline (age 20–24), 22% of men and 26% of women were categorised as having clinically significant depression, 15% of men and 23% of women were categorised as having clinically significant generalised anxiety. Considering comorbidity, 10% of men and 14% of women scored highly for both depression and generalised anxiety.

Table 2 shows the sample baseline characteristics and mean differences for baseline depression and anxiety levels. Greater depression was significantly associated with being female, fewer years of education, being unemployed (compared to working full-time), financial hardship, harmful alcohol use, smoking, lower physical functioning and never entering a partner relationship over the study period. Greater anxiety was significantly associated with being female, fewer years of education, working part-time or not being in the labour force (compared to working full-time), financial hardship, smoking and lower physical functioning. Both baseline depression and anxiety were significantly associated with lower relationship support and greater relationship conflict recorded at follow-up.

**Table 2.** Descriptive statistics, including mean differences and correlations with levels of depression and anxiety at baseline (aged 20–24) (*n* = 1592)

	All participants	Depression (0–9) mean (s.d.) or correlation	Anxiety (0–9) mean (s.d.) or correlation
Gender			
Male	54.5%	2.6 (2.3)*	3.2 (2.6)**
Female	45.5%	3.0 (2.3)	4.3 (2.6)
Age	22.3 (1.5)	−0.03	−0.01
Years of education	14.8 (1.5)	−0.11**	−0.08*
Employment status			
Employed full-time	54.8%	2.7 (2.3)* ^a^	3.4 (2.6)* ^a^
Employed part-time	32.7%	2.8 (2.3)	3.9 (2.7)
Unemployed	5.8%	3.4 (2.6)	4.1 (2.8)
Not in the labour force	6.8%	3.2 (2.3)	4.2 (2.7)
Financial hardship			
No	78.5%	2.5 (2.2)**	3.4 (2.6)**
Yes (often/sometimes)	21.5%	3.8 (2.4)	4.7 (2.7)
Alcohol (hazardous/harmful)			
No	81.8%	2.7 (2.3)*	3.6 (2.7)
Yes	18.2%	3.1 (2.3)	3.8 (2.7)
Smoking status			
No	71.7%	2.6 (2.3)**	3.4 (2.6)**
Yes	28.3%	3.4 (2.3)	4.3 (2.7)
Physical function (0–100)	53.3 (6.7)	−0.14**	−0.16**
In a partner relationship			
Yes (at follow-up)	71.8%	2.7 (2.3)*	3.6 (2.7)
No (never observed)	28.2%	3.1 (2.4)	3.7 (2.7)
Rel. support (0–15)^b^	13.7 (2.1)	−0.19**	−0.17**
Rel. conflict (0–15)^b^	4.6 (3.1)	0.23**	0.18**

*Note*: *p < 0.05. **p < 0.001 notes that this variable is significantly associated with baseline depression or anxiety.

a Post-hoc comparisons (Sidak test) for each level within the ANOVA showed that those who were unemployed had significantly higher depression that those who were employed full-time, and that those who were employed part-time or not in the labour force had significantly higher anxiety than those employed full-time.

b Reported only for participants who reported being in a cohabiting relationship in at least one of the follow-up study waves and reported relationship quality data (*n* = 1030). All covariates taken at baseline, except relationship status and relationship support which use follow-up data.

### Relationship status – findings for baseline depression and anxiety

Results of the logistic regression analyses examining baseline depression and anxiety levels in association with entry (*v*. never entry) into a partner relationship are shown in Table 3. The initial model (1) shows that baseline depression was significantly associated with lower odds of entering a relationship (odds ratio (OR): 0.78, confidence interval (CI): 0.63–0.93). This association remained significant after adjusting for baseline socio-demographic factors, physical functioning and substance use (model 2) (OR: 0.79, CI: 0.64–0.98). All models for baseline anxiety show that levels were not significantly associated with odds of entering a relationship.

**Table 3.** Baseline depression and anxiety predicting odds of ever being (*v*. never being) in a partner relationship (*n* = 1592)

	Model 1: OR (95% CI)	Model 2: OR (95% CI)	Model 1: OR (95% CI)	Model 2: OR (95% CI)
Depression level (baseline)^a^	0.78 (0.63–0.93)*	0.79 (0.64–0.98)*	–	–
Anxiety level (baseline)^a^	–	–	0.91 (0.74–1.13)	0.99 (0.78–1.25)
Drop-out (yes)	0.20 (0.16–0.26)**	0.20 (0.15–0.26)**	0.20 (0.16–0.26)**	0.20 (0.15–0.25)**
Gender (female)		1.07 (0.84–1.37)		1.04 (0.81–1.33)
Age (years)		0.92 (0.85–1.01)		0.93 (0.85–1.01)
Education (years)		1.05 (0.96–1.15)		1.05 (0.97–1.15)
Employ (unemployed)				
Employed full-time		2.20 (1.35–3.58)*		2.26 (1.39–3.68)*
Employed part-time		1.82 (1.10–3.01)*		1.83 (1.11–3.03)*
Not in the labour force		1.32 (0.71–2.46)		1.31 (0.70–2.43)
Financial hardship (yes)		0.92 (0.69–1.23)		0.86 (0.65–1.15)
Alcohol (hazard/harmful)		1.59 (1.14–2.20)*		1.26 (1.12–2.17)
Smoking status (yes)		1.25 (0.94–1.66)		1.22 (0.91–1.61)
Physical function (0–100)		1.02 (1.00–1.04)		1.02 (1.00–1.04)*

*Note*: Logistic regression. *p < 0.05. **p < 0.001.

a The scale scores for depression and anxiety have been rescaled such that a one-point increase on the scales represents the difference between the 25th and the 75th percentiles on the distribution.

### Relationship quality (support and conflict) – findings for baseline depression

Results of the linear mixed model examining baseline depression levels in association with follow-up partner support and conflict are shown in Table 4. The first series of models predicted *relationship support*. The initial models (1 and 2) showed that baseline depression was significantly associated with lower partner support at follow-up – this was the case after adjusting for time-varying covariates. This effect continued to be significant once current levels of depression (at follow-up) were accounted for (model 3, *B*: −0.16, CI: −0.25 to −0.06). The second series of models in Table 4 predicted the level of *conflict in partner relationships*. The initial models (1 and 2) showed that higher baseline depression was significantly associated with higher relationship conflict at follow-up after adjusting for covariates. Models 3 showed that baseline depression continued to predict relationship conflict after adjusting for current levels of depression (model 3, *B*: 0.30, CI: 0.21–0.40).

**Table 4.** Baseline depression predicting relationship quality and conflict at all (3) follow-up waves

	Support from partner (standardised score)			Conflict with partner (standardised score)		
	Model 1: *B* (95% CI)	Model 2: *B* (95% CI)	Model 3: *B* (95% CI)	Model 1: *B* (95% CI)	Model 2: *B* (95% CI)	Model 3: *B* (95% CI)
Depression level (baseline)	−0.32 (−0.44 to −0.25)**	−0.31 (−0.41 to −0.22)**	−0.16 (−0.25 to −0.06)*	0.44 (0.34–0.53)**	0.42 (0.32–0.51)**	0.30 (0.21–0.40)**
Time (wave 2)						
Wave 3	−0.03 (−0.12 to 0.05)	−0.01 (−0.10 to 0.08)	−0.01 (−0.10 to 0.08)	−0.33 (−0.40 to −0.26)**	−0.38 (−0.46 to −0.29)**	−0.37 (−0.46 to −0.29)**
Wave 4	−0.25 (−0.34 to −0.16)**	−0.21 (−0.33 to −0.09)**	−0.21 (−0.33 to −0.010)**	0.23 (0.15–0.32)**	0.13 (−0.02 to 0.24)*	0.14 (0.03–0.24)*
Gender (female)		−0.02 (−0.13 to 0.09)	0.03 (−0.08 to 0.14)		−0.15 (−0.26 to −0.004)*	−0.19 (−0.29 to −0.08)*
Age (years)		−0.02 (−0.06 to 0.02)	−0.02 (−0.06 to 0.02)		−0.03 (−0.06 to 0.01)	−0.02 (−0.06 to 0.01)
Education (years)		0.01 (−0.03 to 0.05)	−0.00 (−0.04 to 0.04)		0.05 (0.02–0.09)*	0.06 (−0.02 to 0.09)*
Children (yes)		−0.13 (−0.24 to −0.31)*	−0.13 (−0.23 to −0.03)*		0.18 (0.09–0.28)**	0.18 (0.09–0.28)**
Employ (unemployed)						
Employed full-time		0.13 (−0.24 to 0.50)	0.11 (−0.26 to 0.48)		−0.53 (−0.87 to −0.19)*	-0.53 (−0.87 to −0.19)*
Employed part-time		0.09 (−0.29 to 0.48)	0.08 (−0.30 to 0.47)		−0.48 (−0.83 to −0.12)*	−0.48 (−0.83 to −0.12)*
Not in the labour force		0.18 (−0.23 to 0.52)	0.17 (−0.23 to 0.57)		−0.56 (−0.93 to −0.19)*	−0.56 (−0.93 to −0.19)*
Financial hardship (yes)		−0.23 (−0.36 to −0.11)**	−0.18 (−0.31 to −0.06)*		0.18 (0.06–0.30)*	0.14 (0.03–0.26)*
Alcohol (hazard/harmful)		0.06 (−0.05 to 0.17)	0.05 (−0.05 to 0.16)		0.01 (−0.09 to 0.11)	0. 02 (−0.08 to 0.11)
Smoking status (yes)		−0.09 (−0.22 to 0.05)	−0.07 (−0.20 to 0.06)		0.14 (0.02–0.27)*	0.13 (0.00–0.25)*
Physical function (0–100)		−0.01 (−0.01 to −0.01)*	−0.01 (−0.02 to −0.01)*		0.00 (−0.00 to 0.01)	0.00 (−0.01 to 0.01)
Current depression (follow-up)			−0.40 (−0.49 to −0.32)**			0.29 (0.22–0.37)**

*Note*: Linear regression. *p < 0.05. **p < 0.001. All covariates are time-varying. The scale scores for baseline and follow-up depression have been rescaled such that a one-point increase on the scales represents the difference between the 25th and the 75th percentiles on the distribution.

No. of observations: Model 1: 1997, Model 2: 1921, Model 3: 1920.

### Relationship quality (support and conflict) – findings for baseline anxiety

Results of the linear mixed model examining baseline anxiety levels in association with levels of subsequent partner support and conflict are shown in Table 5. The initial models (1 and 2) showed that baseline anxiety was significantly associated with lower partner support after adjusting for covariates. The final model showed that higher levels of baseline anxiety continued to be associated with lower partner support (*B*: −0.15, CI: −0.26 to −0.05), after adjusting for follow-up anxiety. The second series of models in Table 5 predicted relationship conflict. The initial models (1 and 2) showed that higher baseline anxiety was significantly associated with higher relationship conflict after covariate adjustment. The final model showed that higher baseline anxiety continued to be associated with higher subsequent conflict after adjusting for follow-up anxiety (*B*: 0.19, CI: 0.09–0.30).

**Table 5.** Baseline anxiety predicting relationship quality and conflict at all (3) follow-up waves

	Support from partner (standardised score)			Conflict with partner (standardised score)		
	Model 1: *B* (95% CI)	Model 2: *B* (95% CI)	Model 3: *B* (95% CI)	Model 1: *B* (95% CI)	Model 2: *B* (95% CI)	Model 3: *B* (95% CI)
Anxiety level (baseline)	−0.32 (−0.42 to −0.22)**	−0.30 (−0.40 to −0.20)**	−0.15 (−0.26 to −0.05)*	0.34 (0.24–0.45)**	0.36 (0.26–0.46)**	0.19 (0.09–0.30)**
Time (wave 2)						
Wave 3	0.03 (−0.12 to 0.05)	−0.01 (−0.11 to 0.08)	−0.00 (−0.09 to 0.08)	−0.33 (−0.40 to −0.25)**	−0.37 (−0.46 to −0.29)**	−0.39 (−0.47 to −0.30)**
Wave 4	−0.24 (−0.34 to −0.15)**	−0.20 (−0.32 to −0.09)*	−0.18 (−0.30 to −0.06)*	0.23 (0.15–0.31)**	0.13 (0.02–0.24)*	−0.10 (−0.01 to 0.21)
Gender (female)		0.02 (−0.09 to 0.14)	0.06 (−0.05 to 0.17)		−0.19 (−0.31 to −0.08)*	−0.24 (−0.35 to −0.13)**
Age (years)		−0.01 (−0.05 to 0.02)	−0.02 (−0.06 to 0.02)		−0.03 (−0.07 to 0.01)	−0.02 (−0.06 to 0.01)
Education (years)		0.01 (−0.03 to 0.05)	0.01 (−0.03 to 0.05)		0.05 (0.01–0.09)*	0.05 (0.01–0.09)*
Children (yes)		−0.14 (−0.24 to −0.04)*	−0.15 (−0.25 to −0.05)*		0.19 (0.09–0.28)**	0.20 (0.11–0.29)**
Employ (unemployed)						
Employed full-time		0.14 (−0.23 to 0.51)	0.10 (−0.27 to 0.47)		−0.54 (−0.88 to −0.20)*	−0.51 (−0.85 to −0.17)*
Employed part-time		0.10 (−0.28 to 0.49)	0.07 (−0.32 to 0.46)		−0.49 (−0.84 to 0.13)*	−0.45 (−0.81 to −0.10)*
Not in the labour force		0.18 (−0.23 to 0.58)	0.14 (−0.26 to 0.55)		−0.56 (−0.93 to −0.18)*	−0.52 (−0.89 to −0.15)*
Financial hardship (yes)		−0.24 (−0.37 to −0.18)**	−0.20 (−0.33 to −0.08)*		0.19 (0.08–0.31)*	0.15 (0.04–0.27)*
Alcohol (hazard/harmful)		0.06 (−0.05 to 0.17)	0.07 (−0.04 to 0.17)		0.01 (−0.09 to 0.11)	0.00 (−0.09 to 0.10)
Smoking status (yes)		−0.10 (−0.24 to 0.03)	−0.07 (−0.20 to 0.07)		0.16 (0.03–0.29)*	0.12 (−0.00 to 0.25)
Physical function (0–100)		−0.01 (−0.01 to −0.01)*	−0.01 (−0.02 to −0.01)*		0.00 (−0.01 to 0.01)	0.01 (−0.00 to 0.01)
Current anxiety (follow-up)			−0.37 (−0.46 to −0.28)*			0.41 (0.33–0.50)**

*Note*: Linear regression. *p < 0.05. **p < 0.001. All covariates are time-varying. The scale scores for baseline and follow-up anxiety have been rescaled such that a one-point increase on the scales represents the difference between the 25th and the 75th percentiles on the distribution.

No. of observations: Model 1: 1997, Model 2: 1921, Model 3: 1920.

### Supplementary analyses

Supplementary analyses were conducted as outlined in the ‘Statistical analyses’ section. All additional analyses essentially mirrored those found in the main analyses. See online Supplementary section and tables for further details.

## Discussion

The current prospective study found that higher depression in early adulthood was associated with a greater likelihood of remaining unpartnered in the future. In addition, for those who did find a partner, depression and anxiety in early adulthood predicted lower relationship support and more conflict. These associations remained significant after adjusting for a range of time-varying socio-economic factors, health behaviours and depression/anxiety levels at follow-up. The enduring association after adjusting for follow-up mental health suggests that past experiences of depression and anxiety matter above and beyond the impact of concurrent mental health problems (which are already known to substantially impact on relationship quality), supporting a greater focus on prevention and early intervention (Jorm, 2014). These findings add to the body of research demonstrating that depression and anxiety in early adulthood have important personal and social costs, not only for the individuals experiencing poor mental health, but also their partners.

Existing epidemiological research exploring early onset depression and anxiety in relation to partner relationships has focused on the consequences for early marriage and teenage parenting (Forthofer *et al*., 1996; Kessler *et al*., 1997; Gotlib *et al*., 1998), and also subsequent separation and divorce (Kessler *et al*., 1998). The current study adds new information, finding that greater levels of depression (but not anxiety) in early adulthood (aged 20–24) are associated with a lower likelihood of finding a long-term partner in the future. This finding is drawn from a sample that had no children or previous partner relationships at baseline (aged 20–24), and therefore (to some extent) confounding concerning premature/adolescent serious partner relationship formation is removed. Overall, the current findings align with research linking depression, social isolation and loneliness (Matthews *et al*., 2016), as well as descriptive (i.e. cross-sectional) studies showing that those with poor mental health are less likely to be in partner relationships (Pearlin and Johnson, 1977; Stack and Eshleman, 1998; Scott *et al*., 2010).

The findings regarding reduced relationship support and increased conflict point towards the pathways *via* which psychiatric symptoms and disorders may result in higher rates of separation and divorce (Kessler *et al*., 1998), offering potential avenues for further research and intervention. Previous research has shown that individuals with any mood disorder were 1.7 times more likely to subsequently separate/divorce and individuals with any anxiety disorder were 1.8 times more likely (Kessler *et al*., 1998). The current findings accord with hypotheses that decline in social support and rise in relationship conflict might explain this association. Most significantly, we find that *early* experiences of depression and anxiety – when young adults are not yet in a significant relationship – can influence the quality of relationships years later.

### Strengths and limitations

The current study has significant strengths, including a large representative community-based sample, the range of socio-economic and health/lifestyle correlates adjusted over time (time-varying), the extensive prospective (rather than retrospective) follow-up period and the exploration of both depression and anxiety symptomology. However, several limitations must be acknowledged. Diagnostic measures were not available to capture diagnosis of a depressive or anxiety disorder at baseline, however, well-validated psychometric measures were used to assess levels of anxiety and depression. Although these scales have validated cut-points to determine a likely diagnosis of generalised anxiety and depressive disorder (Kiely and Butterworth, 2015), in the current analyses they were retained as continuous measures to maximise statistical power. Similarly, measures of relationship support and conflict were self-report and therefore represent participants' perceptions rather than an objective indicator. However, the exposure (baseline mental health) and the outcomes (relationship quality measures) were assessed at different points in time (up to 12 years apart), reducing the influence of ‘common method variable bias’. The current study did not investigate the mechanisms *via* which depression and/or anxiety experienced in early adulthood flow through to impact on relationship quality. These likely include ongoing psychological and interpersonal disruption in accordance with theories and evidence of stress generation (see Hammen, 2005, p. 303 for an overview). Including the idea that depressed or anxious individuals might select themselves into adverse environmental contexts and social relationships (Kendler *et al*., 1999 in Hammen, 2005). Finally, although the prospective study design supports the hypothesis that experiences of depression and anxiety are linked to subsequent poor relationship quality, our findings are not sufficient to conclude this is a causal relationship and more studies are required. For example, it is possible that a negative predisposition or cognitive bias underlies the association between baseline depression and anxiety and follow-up negative perceptions of relationship quality – a plausible alternative explanation for the enduring effect after controlling for current symptomology.

The current study found that future relationship status and quality (i.e. levels of support and conflict) was associated with prior experiences of depression or anxiety in early adulthood. This finding, in conjunction with extant research demonstrating the adverse, far-reaching impacts of poor mental health, supports ongoing calls for a shift in focus from treatment to early intervention and prevention. Preventing mental health problems is critical, not only to reduce the need for (and costs associated with) treatment, but to prevent related social adversity across the lifespan. Engaged, supportive social relationships are an important indicator of quality of life (Helgeson, 2003).

## Supplementary material

For supplementary material accompanying this paper visit http://dx.doi.org/10.1017/S2045796020000530. click here to view supplementary material

## Data

Data from the PATH project can be requested and research conducted in partnership with the PATH Research Committee (see https://rsph.anu.edu.au/research/projects/personality-total-health-path-through-life). The PATH study is jointly conducted by The Australian National University and The University of New South Wales.
