# Supplementary material for: Depression and anxiety in early adulthood: consequences for finding a partner, and relationship support and conflict
Source: Epidemiol Psychiatr Sci. 2020 Jul 15;29:e141. doi: 10.1017/S2045796020000530 (PMC7372160; doi:10.1017/S2045796020000530)
Supplement: Supplementary file 1 [file S2045796020000530sup001.docx]

*Supplementary analyses – Online supplement*

*First relationship only analyses:* Supplementary regression analyses examined the impact of baseline depression and anxiety on future relationship status and levels of partner support and conflict experienced in the *first* marriage or cohabiting relationship recorded at follow-up (i.e. outcome data was recorded only once for participants in the first relationship entered and was then censored). The results closely mirrored those found in the main analyses. Baseline depression levels predicted never being in a relationship at follow-up (Supplementary Table 1), as well as relationship conflict (but not partner support) in the first relationship entered after adjusting for all covariates and current depression (Supplementary Table 2, final Model 3). Baseline anxiety levels predicted follow-up relationship support and conflict after adjusting for all covariates and current anxiety (Supplementary Table 3, final Model 3).

*Transformed outcome analyses:* The relationship quality analyses were repeated using transformed variables for the measures of relationship support and relationship conflict (i.e. taking both the natural logarithm and the square root). The transformed data taking the square root showed some minimal improvement in the skewness of the distributions and when this variable was substituted in the analyses, the core findings essentially replicated those of the original analyses (Adjusted models: depression - positive support (B: -0.01, CI: -0.02 to -.00), conflict (B: 0.06, CI: 0.04 to 0.08); anxiety - positive support (B: -0.01, CI: -0.02 to -0.00), conflict (B: 0.03, CI: 0.02 to 0.05).

*Comorbidity analyses:* The relationship quality analyses in the main paper were repeated using a variable representing depression and anxiety comorbidity. The results showed that in the simple univariate model, experiencing either depression or anxiety at baseline, or both, was significantly associated with poorer relationship support and more relationship conflict at follow-up. These coefficients remained significant after controlling for all socio-economic and health covariates. However, in the final model (3) after controlling for current comorbidity, while experiencing either condition remained significantly associated with lower relationship support and higher conflict (‘1/either’ B: -0.22, CI: -0.36 to -0.08; B: 0.31, CI: 0.17 to 0.33), experiencing both conditions was no longer significant (‘2/comorbid’ B: -0.03, CI: -0.20 to -0.14; B: 0.16, CI: -0.01 to 0.33) (indicating the overwhelming influence of current severe symptomology, and possible over-adjustment).

Supplementary Table 1. Baseline depression and anxiety predicting odds of ever being (vs. never being) in a partner relationship (n=1232).

|  | **Model 1** | **Model 2** | **Model 1** | **Model 2** |
| --- | --- | --- | --- | --- |
| Depression level (0-9) (baseline) | 0.91 (.86, .96)* | 0.93 (0.88, 0.98)* | - | - |
| Anxiety level (0-9) (baseline) | - | - | 0.96 (0.92, 1.01) | 0.98 (0.93, 1.03) |
| Drop-out (yes) | 0.20 (.15, .26)** | 0.21 (0.16, 0.27)** | 0.20 (0.15, .26)** | 0.21 (0.16, 0.27)** |
| Gender (female) |  | 1.08 (.83, 1.42) |  | 1.08 (0.82, 1.41) |
| Age (years) |  | 0.90 (0.82, 0.99)* |  | 0.90 (0.82, 0.99)* |
| Education (years) |  | 1.11 (1.00, 1.22)* |  | 1.11 (1.00, 1.22)* |
| Employ (Unemployed)  *Employed full-time*  *Employed part-time*  *Not in the labour force* |  | 2.33 (1.34, 4.04)*  1.90 (1.08, 3.36)*  1.38 (0.69, 2.78) |  | 2.38 (1.38, 4.13)*  1.93 (1.10, 3.41)*  1.40 (0.70, 2.81) |
| Financial hardship (yes) |  | 0.82 (0.59, 1.13) |  | 0.76 (0.55, 1.05) |
| Alcohol (hazard/harmful) |  | 1.41 (0.99, 2.01) |  | 1.39 (0.98, 1.99) |
| Smoking status (yes) |  | 1.16 (0.85, 1.57) |  | 1.13 (0.83, 1.54) |
| Physical function (0-100) |  | 1.02 (1.00, 1.04) |  | 1.02 (1.00, 1.04) |

Note. Logistic regression. * p<.05. ** p<.001. These supplementary analyse restricted the sample to include those who *never* reported entering a relationship (*n*=449) and those who entered their *first* relationship at one of the three follow-up time-points (*n*=783) (total *n*=1232) (i.e. those who had already been in multiple relationships at follow-up were omitted). In these analyses the measures of depression and anxiety have not been rescaled to the 25^th^ and 75^th^ percentile as in the main paper, thus the range is from 0-9.

Supplementary Table 2. Baseline depression predicting relationship quality and conflict at first follow-up relationship (n=778).

|  | **Support from partner (standardised score)** | | | **Conflict with partner (standardised score)** | | |
| --- | --- | --- | --- | --- | --- | --- |
|  | **Model 1** | **Model 2** | **Model 3** | **Model 1** | **Model 2** | **Model 3** |
| Depression level (0-9) (baseline) | -.08 (-.11, -.05)** | -.07 (-.10, -.04)** | -.02 (-.05, 0.02) | .11 (.08, .14)** | .10 (.07, .13)** | .05 (.02, .09)** |
| Time (wave 2)  *Wave 3*  *Wave 4* | .01 (-.15, .16)  -.21 (-.50, .08) | -.01 (-.17, .14)  -.24 (-.53, .06) | -.02 (-.17, .14)  -.29 (-.58, .00) | -.37 (-.53, -.22)**  .02 (-.26, .31) | -.38 (-.54, -.23)**  -.03 (-.32, .25) | -.38 (-.53, -.23)**  -.02 (-.26, .30) |
| Gender (female) |  | .04 (-.11, .18) | .12 (-.02, .26) |  | -.19 (-.33, -.05)* | -.27 (-.41, -.14)** |
| Age (years) |  | -.03 (-.08, .03) | -.03 (-.08, .03) |  | .03 (-.02, .08) | .03 (-.02, .08) |
| Education (years) |  | .02 (-.04, .07) | -.00 (-.06, .05) |  | .03 (-.02, .09) | .05 (-.00, .10) |
| Employ (Unemployed)  *Employed full-time*  *Employed part-time*  *Not in the labour force* |  | .41 (.04, .77)*  .45 (.07, .82)*  .57 (.12, 1.03)* | .27 (-.07, .64)  .33 (-.04, .69)  .44 (-.01, .88) |  | -.15 (-.51, .21)  .07 (-.30, .44)  .02 (-.42, .47) | -.04 (-.39, .31)  .17 (-.19, .53)  .14 (-.29, .58) |
| Financial hardship (yes) |  | -.26 (-46,-.07)** | -.22 (-41,-.03)* |  | .20 (.01, .38)* | .16 (-.03, .34) |
| Alcohol (hazard/harmful) |  | -.04 (-.23, .15) | -.03 (-.21, .15) |  | .02 (-.15, .20) | .02 (-.15, .19) |
| Smoking status (yes) |  | -.05 (-.22, .12) | -.04 (-.20, .13) |  | .19 (.02, .35)* | .18 (.02, .34)* |
| Physical function (0-100) |  | -.00 (-.02, .01) | -.00 (-.01, .01) |  | .00 (-.01, .01) | -.00 (-.01, .01) |
| Current depression (0-9)  (follow-up) |  |  | -.12 (-.15, -.08)** |  |  | .11 (2.77, .15)** |

Note. Linear Regression. * p<.05. ** p<.001. These supplementary analyse restricted the sample to include those who entered their *first* relationship at one of the three follow-up time-points (*n*=783) (i.e. those who had already been in multiple relationships at follow-up were omitted). In these analyses the measure of depression have not been rescaled to the 25^th^ and 75^th^ percentile as in the main paper, thus the range is from 0-9.

Supplementary Table 3. Baseline anxiety predicting relationship quality and conflict at first follow-up relationship (n=778).

|  | **Support from partner (standardised score)** | | | **Conflict with partner (standardised score)** | | |
| --- | --- | --- | --- | --- | --- | --- |
|  | **Model 1** | **Model 2** | **Model 3** | **Model 1** | **Model 2** | **Model 3** |
| Anxiety level 0-9  (baseline) | -.07 (-.10, -.04)** | -.06 (-.09, -04)** | -.04 (-.07, -.01)* | .07 (.05, .10)** | .07 (.04, .10)** | .03 (.01, .06)* |
| Time (wave 2)  *Wave 3*  *Wave 4* | .01 (-.15, .17)  -.21 (-.50, .08) | -.02 (-.17, .14)  -.24 (-.54, .05) | -.00 (-.16, .15)  -.24 (-.54, .05) | -.37 (-.53, -.21)**  .02 (-.26, .31) | -.38 (-.54, -.22)**  -.03 (-.31, .26) | -.40 (-.55, -.25)**  -.02 (-.30, .26) |
| Gender (female) |  | .09 (-.05, .24) | .13 (-.01, .28) |  | -.24 (-.38, -.10)* | -.31 (-.45, -.17)** |
| Age (years) |  | -.02 (-.07, .04) | -.02 (-.08, .03) |  | .02 (-.03, .08) | .03 (-.02, .08) |
| Education (years) |  | .01 (-.05, .07) | .01 (-.05, .07) |  | .04 (-.02, .09) | .04 (-.02, .09) |
| Employ (Unemployed)  *Employed full-time*  *Employed part-time*  *Not in the labour force* |  | .39 (.02, .75)*  .44 (.08, .82)*  .58 (.12, 1.03)* | .36 (-.00, .73)  .42 (.05, .79)*  .53 (.08, .98)* |  | -.12 (-.49, .24)  .07 (-.30, .44)  .03 (-.42, .48) | -.09 (-.45, .26)  .11 (-.25, .48)  .09 (-.35, .53) |
| Financial hardship (yes) |  | -.27 (-.46,-.08)* | -.22 (-.41,-.03)* |  | .24 (.05, .43)* | .16 (-.03, .35) |
| Alcohol (hazard/harmful) |  | -.03 (-.22, .15) | -.03 (-.21, .15) |  | .01 (-.16, .19) | .01 (-.17, .18) |
| Smoking status (yes) |  | -.05 (-.22, .12) | -.05 (-.22, .12) |  | .22 (.05, .38)* | .21 (.04, .37)* |
| Physical function (0-100) |  | -.00 (-.02, .01) | -.00 (-.02, .01) |  | .00 (-.01, .01) | .00 (-.01, .01) |
| Current anxiety (follow-up) |  |  | -.05 (-.09, -.02)* |  |  | .09 (.06, .12)** |

Note. Linear Regression. * p<.05. ** p<.001. These supplementary analyse restricted the sample to include those who entered their *first* relationship at one of the three follow-up time-points (*n*=783) (i.e. those who had already been in multiple relationships at follow-up were omitted). In these analyses the measure of anxiety has not been rescaled to the 25^th^ and 75^th^ percentile as in the main paper, thus the range is from 0-9.
